# Mannose inhibits *Plasmodium* parasite growth and cerebral malaria development *via* regulation of host immune responses

**DOI:** 10.3389/fimmu.2022.859228

**Published:** 2022-09-23

**Authors:** Li Lv, Zihao Xu, Meichen Zhao, Jian Gao, Rumeng Jiang, Qian Wang, Xiaoyu Shi

**Affiliations:** ^1^Department of Endocrinology and Metabolism, Tianjin Medical University General Hospital, Tianjin, China; ^2^Department of Immunology, School of Basic Medical Sciences, Tianjin Medical University, Tianjin Key Laboratory of Cellular and Molecular Immunology, Key Laboratory of Immune Microenvironment and Diseases of Educational Ministry of China, Tianjin, China; ^3^National Laboratory of Biomacromolecules, Institute of Biophysics, Chinese Academy of Sciences, Beijing, China

**Keywords:** D-mannose, parasitemia, cerebral malaria, macrophage, T cell

## Abstract

D-mannose can be transported into a variety of cells *via* glucose transporter (GLUT), and supraphysiological levels of D-mannose impairs tumor growth and modulates immune cell function through mechanisms such as interference with glycolysis and induction of oxidative stress. Blood-stage *Plasmodium* mainly depends on glycolysis for energy supply and pathological immune response plays a vital role in cerebral malaria. However, it is not clear whether mannose affects malaria blood-stage infection. Here, we fed D-mannose to *Plasmodium berghei*-infected mice and found weight loss and reduced parasitemia without apparent side effects. Compromised parasitemia in C57BL/6 mice was accompanied by an increase in splenic macrophages compared to an untreated group. When mannose was applied to a rodent experimental cerebral malaria (ECM) model, the incidence of ECM decreased. Expression of activation marker CD69 on T cells in peripheral blood and the brain were reduced, and cerebral migration of activated T cells was prevented by decreased expression of CXCR3. These findings suggest that mannose inhibits *Plasmodium* infection by regulating multiple host immune responses and could serve as a potential strategy for facilitating malaria treatment.

## Introduction

The life cycle of *Plasmodium* parasite includes three stages: the mosquito stage, liver stage, and blood stage (1). During the blood stage, parasites invade host red blood cells (RBCs) and reproduce asexually, which causes most of the symptoms of malaria (2). If parasite-infected erythrocytes are sequestered within the blood vessels of the brain, it may lead to a more severe form of disease called cerebral malaria (CM), which is the most serious complication of *Plasmodium falciparum* infection and the most common cause of death (3). CM is difficult to diagnose because the symptoms are similar to many other fever-related diseases. CM is also difficult to treat; even if the patients survive, they may develop long-term neurological and neurocognitive dysfunction (4).

Both RBCs and *Plasmodium* rely heavily on glucose for energy metabolism. RBCs use glucose transporter GLUT1 or GLUT4 for glucose uptake (5), whereas the *Plasmodium* parasite expresses a hexose transporter, termed *Pf*HT in *P. falciparum* (6). The high demand for glucose by infected RBCs (iRBCs) could be beneficial for distinguishing between RBCs and iRBCs for malaria treatment. A similar situation is seen within cancer cells; even under aerobic conditions, glucose still needs to be constantly taken up for glycolysis in cancer cells (7). Glucose transporters are permeable to forms of hexose, such as D-mannose, fructose, and L-ascorbic acid (vitamin C) (8, 9). High-dose vitamin C has been reported to be preferentially absorbed by cancer cells through glucose transporters, subsequently causing oxidative stress and apoptosis (10). A similar strategy has been applied to selective clearance of iRBCs and reduction of CM incidence during *Plasmodium* infection (11).

Recent studies have shown that D-mannose treatment can inhibit tumor growth (12). Upon uptake by GLUT, mannose is quickly converted to mannose-6-phosphate, the intracellular accumulation of which inhibits key enzymes for glucose metabolism (13). High-dose mannose causes oxidative stress in cells (14), and oral administration of mannose in rats treated with thioacetamide (TAA) alleviates intrahepatic oxidative damage (15). In addition, mannose is known to regulate inflammation, particularly by targeting macrophages and T cells (14, 16). Therefore, we hypothesize that mannose treatment inhibits the growth of *Plasmodium* parasites during the blood stage. Here, we show that mannose slows *Plasmodium* growth, not by triggering oxidative stress, but by regulating splenic macrophage population, which serves a critical role in removal of parasitized erythrocytes. Mannose can also prevent CM by inhibiting the migration of activated T cells towards the brain.

## Materials and methods

### Ethics statement

All animal procedures in this study were approved by the Tianjin Medical University (TMU) Ethics Review Committee. This project was conducted according to Laboratory Animal Guidelines for Ethical Review of Animal Welfare (The National Standard of the People’s Republic of China, GB/T 35892-2018) and was approved by the Institutional Animal Care and Use Committee of TMU. *In vitro* cultures of *Plasmodium falciparum* were obtained using human erythrocytes as approved by the TMU Ethics Review Committee and the Tianjin Central Hospital of Gynecology Obstetrics Ethics Review Committee.

### Experimental animals and parasites

Male Wistar rats (80-100 g), 4 to 6-week-old female BALB/c mice, and 6 to 8-week-old female C57BL/6 mice were purchased from SPF Biotechnology Co., Ltd (Beijing, China). All animals were bred at the TMU Animal Care Facility. The blood-stage *P. berghei* ANKA strain and *P. falciparum* 3D7/Dd2/803 strains were stored as stabilates in liquid nitrogen. Parasitemia was counted by light microscope examination of Giemsa-stained thin blood smears.

### *Plasmodium* culture and D-mannose administration

BALB/c mice were infected intravenously with 1×10^4^-1×10^6^ *P. berghei* ANKA-iRBCs. For D-mannose (Sangon Biotech A600554, Shanghai, China) treatment, normal drinking water was exchanged for 20% D-mannose in drinking water (w/v) that was replaced once every 3 days. Mice received mannose by oral gavage (200 µL) every other day from the same stock of 20% mannose in drinking water. These mice were euthanized by cervical dislocation 13 days post-infection (p.i.). *P. falciparum* 3D7/Dd2/803 strains were cultured in human erythrocytes (obtained from Tianjin Central Hospital of Gynecology Obstetrics) in RPMI 1640 medium containing 25 mM HEPES supplemented with 0.5% AlbuMAX II, 100 μM hypoxanthine, and 12.5 μg/mL gentamicin (17). The initial parasitemia in each group was 1%. The cultured parasites were treated daily with 10 mM-50 mM D-mannose for 1 h, which was then replaced with fresh complete medium. The parasitemia of the infected mice and cultured *P. falciparum* was monitored every day.

### Experimental cerebral malaria construction and assessment

Female C57BL/6 mice (6-8 weeks old) were infected intravenously with 1×10^4^ *P. berghei*-iRBCs to construct experimental cerebral malaria (ECM) models (18). Infected mice were treated with 200 μL 20% D-mannose or drinking water by oral gavage every other day starting from day 0, and the drinking water in the experimental groups was changed to 20% mannose. Whether the infected mice developed CM was assessed using the Rapid Murine Coma and Behavior Scale (RMCBS) every 24 hours from 6 days p.i. as described previously (19). Mice that developed CM died or were euthanized when found moribund. At the end point of this experiment, the remaining mice were euthanized by cervical dislocation. The integrity of the blood-brain barrier (BBB) was determined by Evans Blue staining, and Evans Blue extravasation of the brain was calculated as milligrams of Evans Blue dye per gram of brain tissue as described previously (20).

### Intracellular oxidative stress analysis

*P. berghei-*parasitized rat erythrocytes were purified by density gradient centrifugation using Histodenz (Sigma-Aldrich D2158, St. Louis, Missouri, US). The superoxide levels of iRBCs/RBCs were determined by detecting the mean fluorescence intensity of DHE (Beyotime, Shanghai, Beijing, China) and MitoSOX Red (Invitrogen, Carlsbad, CA, United States) by flow cytometry. Isolated iRBCs/RBCs were treated with 10 mM-30 mM D-mannose for 3 h, and then stained with 10 μM DHE for 30 min and 5 μM MitoSOX Red for 15 min. Menadione or H_2_O_2_-treated cells were used as positive controls. Stained cells were analyzed on a BD FACSCanto II Flow Cytometer (BD Biosciences, San Jose, CA, US) and data analyzed by FlowJo software (TreeStar, Ashland, OR, US).

### Annexin V apoptosis assay

Eryptosis of RBCs and iRBCs was detected by the Annexin V-FITC Apoptosis Detection Kit (Thermo Fisher Scientific, US) according to the manufacturer’s instructions. Rat RBCs and purified *P. berghei*-iRBCs were treated with 10-30 mM mannose for 3 h and stained with Annexin V-FITC before flow cytometry. Cells treated with 10 mM vitamin C for 1 h were used as positive controls. Cultured *P. falciparum* was treated with 10-30 mM mannose for 24 h and analyzed by the Annexin V apoptosis assay.

### TUNEL assay

TUNEL assays were performed using the CoraLite^®^488 TUNEL Fluorescence Assay Kit (Proteintech, US) in accordance with the manufacturer’s instructions. Parasite-infected erythrocytes treated or not treated with mannose and DNase I were fixed with 4% formaldehyde for 30 min and permeabilized with 0.2% Triton X-100 for 2 min. The cells were labeled with TUNEL reaction mix for 1 h at 37°C and analyzed by flow cytometry. The percentage of apoptotic cells was determined as the proportion of TUNEL-positive cells among total cells. DNase I-treated cells were used as the positive control.

### Isolation and analysis of the leukocyte population

To determine the macrophage population in mouse spleens, the organ was harvested on the indicated days and spleen single-cell suspensions prepared by homogenization through a 70-μM cell strainer (BD Falcon Bioswisstec, Switzerland). Erythrocyte lysis was performed to acquire splenocytes. Peripheral blood was collected by cardiac puncture and anticoagulated with heparin to measure CD4^+^ and CD8^+^ T cells. Peripheral blood mononuclear cells were isolated using the Mouse Peripheral Blood Mononuclear Cell Isolation Kit (Solarbio, China) according to the manufacturer’s protocol. To determine the infiltration of T cells into the brain, brains were dissected on the indicated days after perfusion with 1×PBS, and brain mononuclear cells were isolated following a previously reported procedure (21). The total cell concentrations of splenocytes or mononuclear cells from the brain and peripheral blood were calculated using a hemocytometer, and cell viability was evaluated using Trypan blue staining.

The following antibodies purchased from BioLegend, eBioscience, or BD were used: CD45-PerCP (30-F11), CD11b-APC (M1/70), CD11b-PerCP/Cy5.5 (M1/70), F4/80-FITC (BM8), F4/80-APC (BM8), Ly-6G-PE (1A8), MHC Class II-PE (M5/114.15.2), CD206-PE/Cyanine7 (C068C2), CD3e-FITC (145-2C11), CD4-PE (RM4-5), CD4-PerCP/Cy5.5 (RM4-5), CD8-APC (53-6.7), CXCR3-PE (CXCR3-173), CD69-PE/Cyanine7 (H1.2F3), and CD16/32 (93). All cell preparations were incubated with anti-mouse CD16/32 on ice for 15 min before staining, and then incubated with cocktails of mAbs in flow cytometry buffer. Stained cells were analyzed using a BD FACSCanto II Flow Cytometer and data analyzed using FlowJo software.

### Macrophage phagocytosis assay

The phagocytosis assay was performed according to the protocol described by Yan et al. (22). Mouse spleen mononuclear cells were isolated and cultured in DMEM complete media with 10% FBS (D10) in a 6-well plate. Cells were incubated at 37°C for 1 hour to allow the macrophages to adhere, and non-adherent cells were subsequently removed by washing. IgG-opsonized GFP-expressing *Escherichia coli (E. coli)* was used for the phagocytosis of splenic macrophages. After washing the cultured cells, 2 mL fresh D10 containing *E. coli* (~100 bacteria per cell) was added to each well. Bacterial attachment was synchronized by spinning the 6-well plate at 500 rpm for 5 min at room temperature (RT). The cells were incubated at 37°C for 1 hour to allow bacterial uptake by macrophages. We added 200 μL 0.25% Trypsin-EDTA to the cells and incubated for 10 min at RT to remove the residual *E. coli* at the cell surface. The plate was washed three times with PBS and 0.5 mL 5 mM EDTA added to each well to detach the cells for 5 min, and then 2 mL D10 was used to stop digestion. The cells were harvested by spinning and the cells washed with PBS. The cells were then ready for flow cytometric analysis to evaluate the phagocytotic capacity.

### RNA extraction and real-time PCR

Total RNA was extracted from tissues using TRIzol (Invitrogen, Carlsbad, CA, US) according to the manufacturer’s instructions, and cDNA was synthesized using a HiFiScript cDNA Synthesis kit (CoWin Biosciences, Beijing, China). The UltraSYBR Mixture kit (CoWin Biosciences, Beijing, China) was used for single-step real-time PCR reactions. For PCR, an initial cycle of 95°C for 10 min was followed by 40 cycles of 95°C for 15 sec and 60°C for 1 min. A thermal denaturation protocol was then used to generate the dissociation curves to verify the amplification specificity (a single cycle of 95°C for 15 s, 60°C for 60 s, 95°C for 15 s, and 60°C for 10 s). The reactions were performed on a LightCycler ^®^ 96 System (Roche, Basel, Switzerland). Changes in mRNA levels were quantified using the 2^-ΔΔCT^ method with β-actin mRNA as the internal control. Primers for β-actin were 5’-GGCTGTATTCCCCTCCATCG-3’ (forward) and 5’-CCAGTTGGTAACAATGCCATGT-3’ (reverse). Primers for *P. berghei* 18S rRNA were 5’-AAGCATTAAATAAAGCGAATACATCCTTAC-3’ (forward) and 5’-GGAGATTGGTTTTGACGTTTATGTG-3’ (reverse). Primers for IFN-γ were 5’-CCCTCACACTCAGATCATCTTCT-3’ (forward) and 5’-TGCTACGACGTGGGCTACAG-3’ (reverse). Primers for TNF-α were 5’-ATGAACGCTACACACTGCATC-3’ (forward) and 5’-CCATCCTTTTGCCAGTTCCTC-3’ (reverse).

### Assessment of mouse health status and serum biochemical indices

Infected and uninfected mice were treated with drinking water or 20% D-mannose and their body weight and food intake monitored daily. Average daily food consumption was expressed as grams of total food eaten per total weight of all animals in the cage. Blood samples were collected by retro-orbital bleeding after 20 days of treatment for normal BALB/c mice or on day 7 p.i. for infected C57BL/6 mice. Serum was harvested to detect biochemical indices using a Roche P800 Clinical Biochemistry Analyzer (F. Hoffmann-La Roche Ltd., Basel, Switzerland), including alanine aminotransferase (ALT), aspartate transaminase (AST), total bilirubin (TBIL), urea (BUN), and creatinine (CREA).

### Statistical analysis

Data are presented as mean ± SD. The unpaired *t* test or Mann-Whitney *U* test was used to compare two different groups. One-way ANOVA (with Tukey’s multiple-comparison post-test) or Kruskal-Wallis ANOVA was used for comparisons between more than two groups. Two-way ANOVA with Tukey’s multiple comparisons test was performed to compare the effect of multiple levels of two factors. The cumulative survival rates of mice between two groups were plotted by the Kaplan-Meier method and compared using the log-rank test. *P* < 0.05 was considered significant. All statistical analyses were performed using GraphPad Prism 7.

## Results

### Mannose inhibits rodent and human *Plasmodium* growth

**Figure 1:**
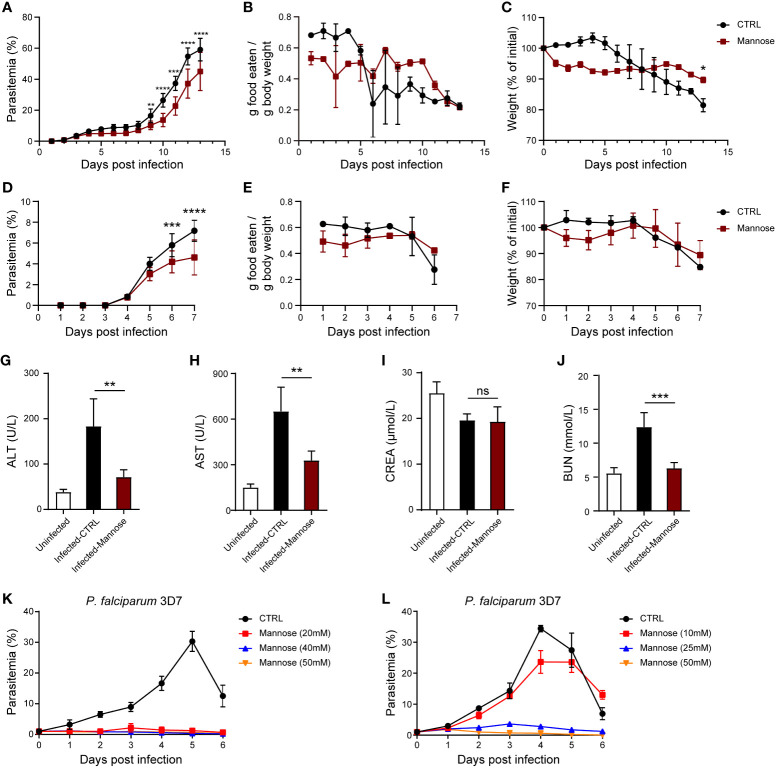
Mannose inhibits *Plasmodium* growth and is well tolerated in *P. berghei*-infected mice **(A)** Mannose (20%) inhibited *P. berghei* ANKA growth in BALB/c mice (n = 10 mice/group). Drinking water or mannose was supplemented by oral gavage every other day after infection. **(B)** Food intake by the infected mice in **(A)**. The average daily food consumption in the indicated group was expressed as grams of food eaten per total animal weight in that cage (5 mice/cage). **(C)** Daily body weight of the infected mice in **(A)** expressed as a percentage of the starting weight. **(D)** Mannose (20%) inhibited *P. berghei* ANKA growth in C57BL/6 mice (n = 8/group). Drinking water or mannose was supplemented by oral gavage every other day after infection. **(E)** Food intake by the infected mice in **(D)** (4 mice/cage). **(F)** Body weight of the infected mice in **(D)**. **(G, H)** Treatment with mannose significantly decreased serum ALT and AST levels in infected C57BL/6 mice 7 days post-infection (p.i.; n = 4/group). **(I, J)** Treatment with mannose alleviated kidney dysfunction in infected C57BL/6 mice 7 days p.i. (n = 4/group). **(K, L)** D-mannose inhibited blood-stage *P. falciparum* 3D7 growth *in vitro*. *P. falciparum* 3D7 was cultured *in vitro* with a starting parasitemia of 1% on day 0 (n = 3/group). The indicated dose of D-mannose was added to the media for 1 hour from day 0 and parasitemia determined daily before mannose treatment. CTRL vs. Mannose (20/40/50 mM): *P* < 0.0001; CTRL vs. Mannose (25/50 mM): *P* < 0.0001; CTRL vs. Mannose (10 mM): ns. All data are presented as mean ± SD and were analyzed by the Kruskal-Wallis ANOVA or two-way ANOVA with Tukey’s multiple comparisons test. *P < 0.05, **P < 0.01, ***P < 0.001, ****P < 0.0001, and ns, not significant.

To test whether mannose inhibits the growth of blood-stage parasites, we infected BALB/c mice with *P. berghei* ANKA, fed them drinking water containing 20% mannose daily, and treated them with 200 μL 20% mannose by oral gavage every other day (qod) from the day of infection. The same treatments have been used in mice for tumor inhibition (12) and a single oral gavage of 200 μL 20% mannose in mice elevates the serum concentration of mannose to ~3 mM (12). Infected mice not treated with mannose exhibited a typical parasite growth pattern over 13 days (23), whereas mice treated with mannose exhibited reduced parasitemia during both the initial peak and the latter proliferation (**Figure 1A**). Infected mice not treated with mannose exhibited a gradual decline in body weight and food intake, which correlated with sickness-related anorectic behavior (**Figures 1B, C**). In contrast, these aspects of infected and treated mice exhibited a slight decrease in the early phase of infection while remained relatively constant during the later course of infection (**Figures 1B, C**). These results suggest that mannose treatment delays the course of *Plasmodium* parasite infection even though complete elimination is not achieved.

Next, we tested whether mannose is still effective when infection has been established. When mannose treatment was started 5 days p.i., when parasitemia was evident, the inhibition of parasite growth was still visible in the later stages of infection but was less effective than treatment starting from 2 days p.i. (**Supplementary Figure 1A**). We also analyzed mannose efficacy in preventing *Plasmodium* infection by administering mannose 10 days prior to parasite infection. We observed no difference in the progression of infection compared to infected mice that were treated starting 1 day p.i. (**Supplementary Figure 1B**). These results suggest that mannose treatment is more efficient when administered at an early stage of infection but does not offer additional protection upon pre-treatment.

We also tested whether mannose treatment is sensitive to the genetic backgrounds of host mice. C57BL/6 mice are commonly used to generate the ECM model. We found growth inhibition of blood-stage parasite by mannose (**Figure 1D**). Similar patterns of food intake and body weight were seen in the treated and untreated groups compared to the BALB/c groups (**Figures 1E, F**). In addition, liver and kidney dysfunction observed during infection were reduced 7 days p.i. in mannose-treated mice (**Figures 1G–J**). These results indicate that mannose is effective in inhibiting *Plasmodium* growth and alleviating symptoms in C57BL/6 mice.

Finally, we tested whether mannose works on *P. falciparum*. Gonzalez et al. reported that 25 mM mannose impaired the growth of several kinds of cancer cells *in vitro* (12). Therefore, we added mannose to *in vitro* cultured *P. falciparum* for 1 h each day. Parasite growth was efficiently inhibited by the addition of high dose of mannose (≥ 20 mM) (**Figure 1K**) but was not effective at 10 mM (**Figure 1L**). We also tested the efficacy of mannose treatment against drug-resistant *P. falciparum* strains. Similar results were obtained when high dose of mannose (≥ 20 mM) was used in chloroquine-resistant *P. falciparum* Dd2 and artemisinin-resistant *P. falciparum* 803 strains (**Supplementary Figures 1C, D**). These results confirm that mannose treatment inhibits the growth of *P. falciparum in vitro* with high dose of mannose (≥ 20 mM) and is effective in previously known drug-resistant strains.

### Mannose-induced *Plasmodium* inhibition is not related to oxidative stress

High-dose mannose causes oxidative stress in cells (14). To determine whether mannose treatment triggers oxidative stress in iRBCs, isolated *P. berghei* ANKA-infected rat RBCs and cultured *P. falciparum* 3D7 were incubated with 10-30 mM mannose for 3 h and subjected to superoxide measurements. Surprisingly, accumulation of reactive oxygen species (ROS), as monitored by DHE or MitoSOX, was not detected with increasing concentrations of mannose (**Supplementary Figures 2A–C**). The combination of mannose with chemotherapeutic drugs has been reported to trigger cancer cell death by potentiating the intrinsic pathway of apoptosis (12). However, *in vitro* treatment with mannose did not cause eryptosis in erythrocytes infected by *P. berghei* ANKA or *P. falciparum* 3D7 (**Supplementary Figures 2D, E**) or apoptosis in *P. berghei* parasites (**Supplementary Figure 2F**). In contrast, vitamin C treatment consistently triggered eryptosis in iRBCs (**Supplementary Figure 2D**) as reported previously (11). Taken together, these results suggest that mannose-induced *Plasmodium* inhibition is less likely attributed to increased oxidative stress or the induction of apoptosis.

### Mannose prevents onset of experimental cerebral malaria

**Figure 2:**
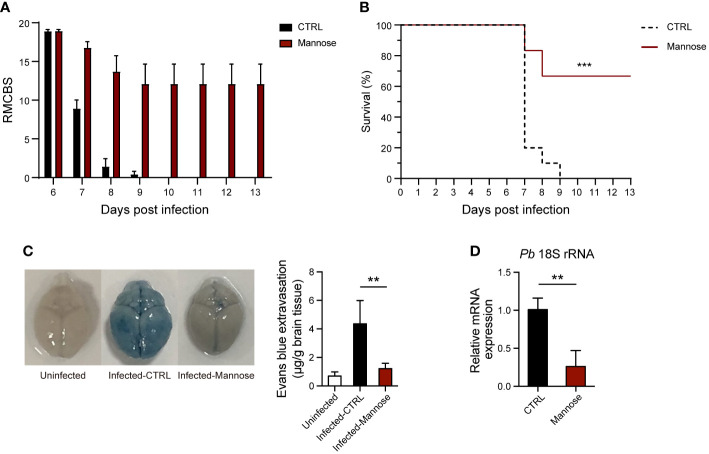
Mannose inhibits ECM development and parasite sequestration in the brain. **(A)** C57BL/6 mice infected with *P. berghei* were evaluated daily from day 6 post-infection (p.i.) using the RMCBS (n = 10-12/group). Scores significantly declined when the mice developed ECM. **(B)** D-mannose treatment significantly inhibited cerebral malaria development. CTRL vs. Mannose (+): *P* < 0.001 (Log-rank test). **(C)** Mannose treatment protected BBB integrity. Left: Representative images of brains from mice injected with Evans Blue to detect BBB leakage. Right: Quantification of Evans Blue dye in brains of mice in three groups (n = 3-5/group). **(D)** Mannose treatment inhibited parasite sequestration in the brain. Relative *P. berghei* 18S rRNA expression in the brain was determined by real-time PCR 7 days p.i. (mouse β-actin as internal control, n = 4/group). Data are presented as mean ± SD and were analyzed by the Kruskal-Wallis ANOVA. **P < 0.01, ***P < 0.001.

The plasma concentrations of mannose in *in vivo* experiments are much lower than the dosage used in *in vitro* growth inhibition assay, suggesting different mechanisms of parasite-growth inhibition between *in vivo* and *in vitro* experiments. Hence, we investigated the effect of mannose on CM, a life-threatening form of malaria infection. Mannose treatment reduced the incidence of ECM in *P. berghei*-infected C57BL/6 mice based on the RMCBS (**Figure 2A**) and increased the survival rate to ~70% compared to the untreated group (**Figure 2B**). Consistently, BBB leakage was observed in infected mice, but the integrity of the BBB was protected by mannose treatment (**Figure 2C**). Parasite sequestration in the brains of infected mice was also diminished in the mannose-treated group when detected 7 days p.i. (**Figure 2D**). These results suggest that mannose treatment decreases the incidence of ECM.

Mannose treatment appears beneficial in eliminating CM, but the required dosage of administration is relatively high. Thus, we analyzed the general conditions of healthy mice treated with the same amount of mannose. Mannose treatment decreased the body weight of mice over a 7-day period (**Supplementary Figure 3A**). Similarly, food intake per cage was reduced upon mannose treatment (**Supplementary Figure 3B**). Loss of weight and food intake suggests that high-dose supplementation of mannose influences host metabolism. Therefore, we also measured biochemical indices in sera collected from the three groups that were treated for 20 days to assess their liver and kidney functions. ALT and AST, which represent the general liver condition, were not significantly changed among the groups (**Supplementary Figure 3C**). The same results were obtained when CREA and BUN, which indicate kidney function, were tested (**Supplementary Figure 3D**). Collectively, these results suggest that, although body weight and food intake were affected, the overall health condition of the mice was not compromised by treatment with 20% mannose.

### Mannose enhances splenic macrophage accumulation in *P. berghei*-infected C57BL/6 mice

**Figure 3:**
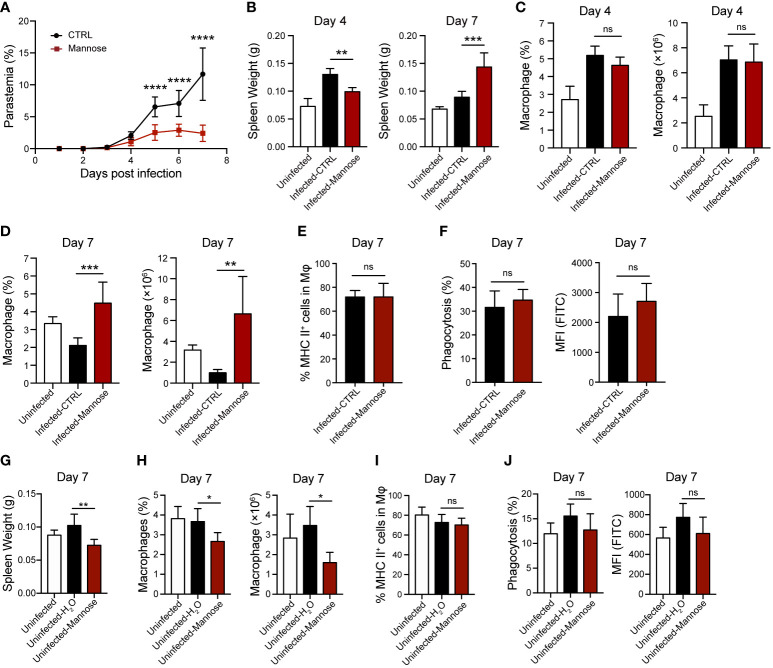
Mannose treatment increases splenic macrophage population in *P. berghei*-infected C57BL/6 mice. **(A)** Parasitemia of *P. berghei-*infected C57BL/6 mice (n = 8/group). Two-way ANOVA with Tukey’s multiple comparisons test was used to analyze differences in parasitemia between the control and mannose-treated groups. **(B)** Spleen weight in C57BL/6 mice (n = 10/group) 4 and 7 days post-infection (p.i.). The weights of spleens harvested from uninfected mice (n = 5/group) are shown as blank controls. **(C)** The frequency and number of splenic macrophages (CD45^+^ F4/80^+^ CD11b^+^) in the infected C57BL/6 mice 4 days p.i. (n = 5/group) and the uninfected mice (n = 5/group) were quantified. **(D)** The frequency and number of splenic macrophages (CD45^+^ F4/80^+^ CD11b^+^) in the infected C57BL/6 mice 7 days p.i. (n = 6/group) and the uninfected mice (n = 5/group) were quantified. **(E)** Quantification of the ratio of M1 macrophages (MHC II^+^) to total splenic macrophages in the infected mice 7 days p.i. **(F)** Phagocytic capacity of splenic macrophages from the infected mice 7 days p.i. The percentage of phagocytosing FITC^+^ macrophages and mean fluorescence intensity (MFI) values. **(G)** Spleen weight in the uninfected C57BL/6 mice on day 7 (n = 5/group). **(H)** The frequency and number of splenic macrophages (CD45^+^ F4/80^+^ CD11b^+^) in the uninfected C57BL/6 mice on day 7 (n = 5/group). **(I)** Quantification of the ratio of M1 macrophages (MHC II^+^) to total splenic macrophages in the uninfected mice on day 7. **(J)** Phagocytic capacity of splenic macrophages from the uninfected mice on day 7. The percentage of phagocytosing FITC^+^ macrophages and MFI values. Data are presented as mean ± SD. Analyses were carried out by one-way ANOVA followed by Tukey’s multiple comparison test or unpaired *t* test. *P < 0.05, **P < 0.01, ***P < 0.001, ****P < 0.0001, and ns, not significant.

Reduced parasitic burden in the peripheral blood and sequestration of iRBCs in the brain suggest enhanced parasite clearance in the mannose-treated group. Therefore, we analyzed the splenic macrophage population, which is mainly responsible for early parasite clearance (24), in ECM-susceptible C57BL/6 mice 4 and 7 days p.i. Detectable differences in parasitemia between untreated and treated groups of infected mice were observed from 5 days p.i. (**Figure 3A**). A swollen spleen, as measured by weight, was readily detected after infection. Comparing mannose-treated and untreated mice, spleen weights were lower 4 days p.i. but higher 7 days p.i. (**Figure 3B**). The percentage and absolute number of F4/80^+^ CD11b^+^ macrophages in the total population of CD45^+^ splenocytes were not remarkably different between the two infected groups 4 days p.i. (**Figure 3C** and **Supplementary Figure 4A**) but significantly increased in the mannose group 7 days p.i. (**Figure 3D** and **Supplementary Figure 4B**), consistent with the increased spleen weight (**Figure 3B**). The classification of splenic macrophages was subsequently analyzed and M1 macrophages (MHC II^+^ cells) found to comprise > 60% of total macrophages in the spleen. No significant differences in the macrophage population were observed between the mannose-treated and non-treated groups (**Figure 3E**). We also evaluated the phagocytotic capacities of the splenic macrophages, with or without mannose treatment, and found no obvious distinctions (**Figure 3F**). These results suggest that mannose promotes splenic macrophage accumulation which may participate in the removal of circulating parasites, but less likely influences their subtypes and phagocytosis.

Next, we tested the effects of mannose on splenic macrophages in uninfected C57BL/6 mice. As previously suggested, mannose treatment significantly decreased spleen weight and macrophage levels on day 7 (**Figures 3G, H** and **Supplementary Figures 4C–F**). Similar to infected mice, mannose had no effect on macrophage subtypes and their phagocytotic capacities in the uninfected mice (**Figures 3I, J** and **Supplementary Figures 4G, H**). These results suggest that mannose may play a distinct role in regulating splenic macrophage levels in *P. berghei*-infected C57BL/6 mice compared to uninfected, normal mice.

We also analyzed splenic macrophages in infected BALB/c mice. The spleen weight remained the same on 4 days p.i. and did not increase with mannose treatment at 7 days p.i. Instead, it slightly decreased on day 7, and the macrophage population showed no difference between the two infected groups 4 and 7 days p.i. (**Supplementary Figure 5**). These results suggest that mannose action is different in infected C57BL/6 mice than in BALB/c mice, likely due to differences in their genetic backgrounds and immune responses that are yet to be identified.

### Mannose regulates T-cell activation and brain migration

**Figure 4:**
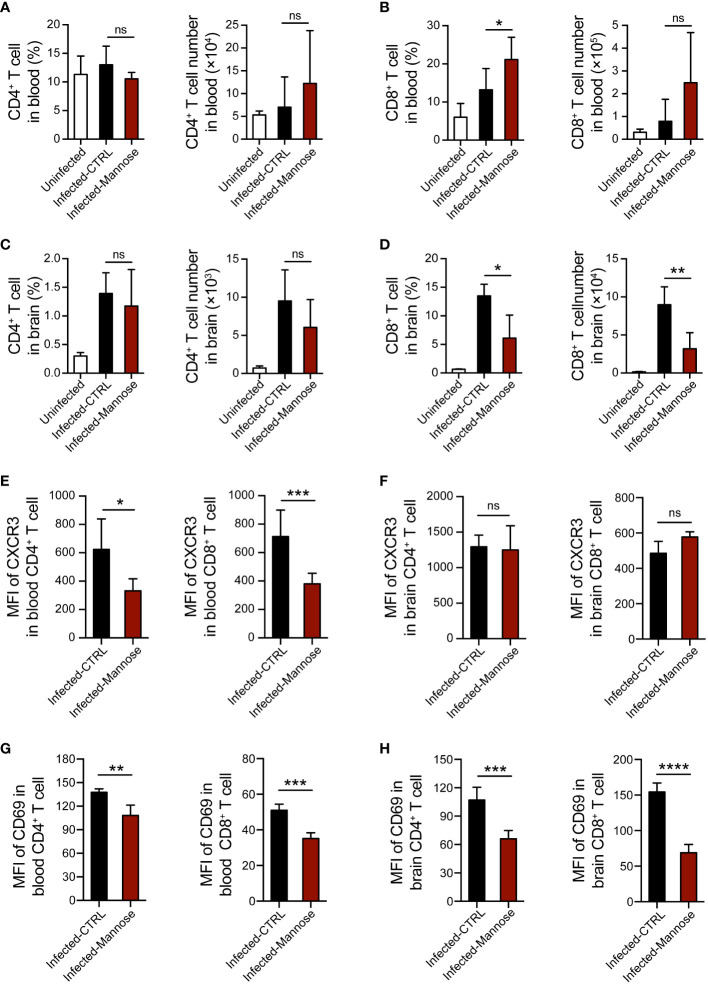
Mannose treatment suppresses T-cell activation and migration into the brain in infected C57BL/6 mice. **(A, B)** Mannose treatment promoted the T-cell response in peripheral blood from infected C57BL/6 mice. The frequency and number of CD4^+^ T cells **(A)** and CD8^+^ T cells **(B)** in peripheral blood mononuclear cells from uninfected (n = 4/group) and infected mice 7 days p.i. (n = 5-9/group) were quantified. **(C, D)** Mannose treatment influenced T-cell infiltration in the brains of infected C57BL/6 mice. The frequency and number of CD4^+^ T cells **(C)** and CD8^+^ T cells **(D)** in brain mononuclear cells from uninfected (n = 3/group) and infected mice 7 days p.i. (n = 3-5/group) were quantified. **(E, F)** The mean fluorescence intensity (MFI) of CXCR3 expression on CD4^+^ T cells and CD8^+^ T cells in the peripheral blood **(E)** and brains **(F)** of infected mice 7 days p.i. **(G, H)** The MFI of CD69 expression on CD4^+^ T cells and CD8^+^ T cells in the peripheral blood **(G)** and brains **(H)** of infected mice 7 days p.i. Data are presented as mean ± SD and were analyzed by unpaired *t* test or Mann-Whitney *U* test. *P < 0.05, **P < 0.01, ***P < 0.001, ****P < 0.0001, and ns, not significant.

T-cell migration towards the brain and secretion of inflammatory factors have been shown to play important roles in CM pathogenesis (25). To investigate whether mannose treatment modulates the T-cell response during ECM development, we quantified the CD4^+^ and CD8^+^ T cell populations in peripheral blood and brain 7 days p.i., when CM-related neurological symptoms are most prominent. Circulating T cells were significantly increased upon infection compared to uninfected mice. In infected mice, CD8^+^ T cells slightly increased in the mannose-treated group, whereas CD4^+^ T cells did not change (**Figures 4A, B** and **Supplementary Figures 6A, B**). In contrast, the frequency and number of CD8^+^ T cells sequestered in the brain were significantly decreased after mannose treatment (**Figures 4C, D** and **Supplementary Figures 6C, D**). Because CXCR3 expression on T cells is associated with the migration of T cells to the brain (26), we tested CXCR3 expression on CD4^+^ and CD8^+^ T cells from the peripheral blood and brain. In peripheral blood, we noted a decrease in CXCR3 expression on both CD4^+^ and CD8^+^ T cells upon mannose treatment (**Figure 4E**). In the brain, we observed no detectable differences in CXCR3 expression on infiltrated T cells between the control and mannose-treated groups (**Figure 4F**). In addition, the expression of activation marker CD69 on T cells was significantly reduced in the peripheral blood and brain in the mannose-treated groups (**Figures 4G, H**).

The activation of T cells infiltrated in brain affects their secretion of cytokines, including IFN-γ and TNF-α, which play essential roles in ECM pathogenesis (27, 28). Therefore, we tested the mRNA expression levels of IFN-γ and TNF-α in brain tissues and found these two cytokines were significantly decreased in the infected mice treated with mannose (**Supplementary Figures 6E, F**). Taken together, these data suggest that mannose likely suppress ECM development by regulating T-cell activation and migration to the brain in mice infected with *P. berghei* ANKA.

## Discussion

Our results reveal an activity of mannose to inhibit blood-stage *Plasmodium* parasites. The chemical similarity between glucose and mannose allows mannose to cross iRBC membranes through GLUT1/4 and hexose transporter on the surface of parasites (29, 30). Based on the application of mannose in killing cancer cells (12, 14), we initially expected induction of oxidative stress and apoptosis/eryptosis in iRBCs and parasites by mannose. However, our results indicate that mannose mainly targets the immune system for *P. berghei* inhibition. The mechanism remains elusive, as the immune system has little effect in *in vitro* culture.

Spleen clearance of malaria parasites likely accounts for reduced parasite burden and tissue sequestration. Mannose treatment can reduce parasitemia in both infected BALB/c and C57BL/6 mice, but the splenic macrophage variations in the two mice exhibit different patterns. In ECM-susceptible C57BL/6 mice, the splenic macrophages were significantly elevated 7 days p.i. with mannose treatment. In contrast, in ECM-resistant BALB/c mice, the splenic macrophage population exhibited no variations 7 days p.i. However, mannose treatment decreased the splenic macrophage levels in uninfected C57BL/6 mice. Our results suggest that the regulation of macrophages by mannose differs in mice with distinct genetic and immune backgrounds and *Plasmodium* infection status, and mannose plays a more vital role in immune regulation in ECM-susceptible C57BL/6 mice only when infection is present. The mannose-induced accumulation of macrophages in the spleens of infected C57BL/6 mice allows for increased clearance of iRBCs and reduced parasite burden, the direct evidence of which still needs further exploration.

Mannose treatment also reduces the incidence of ECM in a mouse model. In this case, growth inhibition is less likely the cause because ECM development is less sensitive to exact parasitemia and a higher inoculum dose has been reported to decrease the incidence of ECM (18, 31). Mannose is used as a non-antibiotic treatment of recurrent urinary tract infection by preventing bacterial adhesion to the urothelium (32). It is also reported to play a role in modulating regulatory T-cell differentiation and lipopolysaccharide-induced macrophage activation, suppressing T cell-mediated immunopathology, lipopolysaccharide-induced endotoxemia, and dextran sulfate sodium-induced colitis (14, 16). CM is considered to be an immune-mediated disease. The hallmark of ECM pathogenesis is the activation and migration of T cells to brain tissue, where iRBCs are sequestered (33). These brain-infiltrated T cells act on activated cerebrovascular endothelial cells by triggering apoptosis and subsequent BBB breakage (34). During the process, inflammatory factors, such as IFN-γ and TNF-α, are frequently secreted and participate in ECM development (35, 36). We found that CD8^+^ T-cell infiltration, activation of both CD4^+^ and CD8^+^ T cells, and the expression of IFN-γ and TNF-α in the brain were reduced in the ECM model upon mannose treatment. These findings suggest that mannose-administration may decrease the incidence of ECM *via* modification of host immune response. Although the mechanisms underlying the inhibited T-cell activation and brain infiltration were not directly addressed in this study, we confirmed that mannose treatment resulted in pleiotropic alterations. Future studies will be required to clarify whether mannnose acts directly on T cells or just regulates T cell response through an indirect way, such as enhanced protective immunity induced by low-level malaria infections.

We checked the safety of the mannose dosage used in the infected mice and normal uninfected mice. Interestingly, the body weight and food intake of normal mice declined after a 7-day mannose treatment, whereas weight loss and the sickness-related anorectic behavior of the infected mice were mitigated after mannose treatment. These results indicate that supplement of high dose mannose may have an effect on the host metabolism. Several studies have focused on the effect of host metabolism on *Plasmodium* infection and found that host metabolism affects malaria tolerance; host glucose utilization, caloric restriction, or high-fat diet could impact malaria burden or cerebral malaria development (37–39). Whether similar mechanisms apply to mannose-induced *Plasmodium* inhibition remains to be tested. Nevertheless, a long period of mannose supplementation had no apparent toxicity. Similarly, no major side effect was reported in previous *in vivo* studies of mannose administration (12). The physiological concentration of mannose in the serum of humans and mice is approximately 100 μM (40), whereas a single oral gavage of 200 μL 20% mannose in mice elevates the serum concentration of mannose to ~3 mM (12). Mannose has been used clinically to treat urinary tract infection (41), but the dosage is much lower than used here, suggesting different mechanisms. In a study of patients with carbohydrate-deficient glycoprotein syndrome type I, mannose was administered by continuous intravenous infusion for 3 weeks, reaching a stable serum level of up to 2 mM, and was well tolerated (42). Whether mannose treatment prevents CM in humans is not clear. Based on the current dosage and efficacy, it is less likely that mannose treatment will be clinically acceptable for first-line therapy of malaria. Nevertheless, the drug resistance problem of current anti-malarial treatments urges alternative solutions. As such, mannose would be a decent candidate for combined treatments considering the mechanism of action does not overlap with at least chloroquine and artemisinin.

## Data availability statement

The original contributions presented in the study are included in the article/**Supplementary Material**. Further inquiries can be directed to the corresponding authors.

## Ethics statement

The studies involving human participants were reviewed and approved by Tianjin Medical University (TMU) Ethics Review Committee and Tianjin Central Hospital of Gynecology Obstetrics Ethics Review Committee. The patients/participants provided their written informed consent to participate in this study. The animal study was reviewed and approved by Tianjin Medical University (TMU) Ethics Review Committee.

## Author contributions

XS and QW conceived and designed the study. LL, ZX, MZ, JG, XS and RJ acquired and analyzed the data. XS, MZ and RJ drafted the manuscript. QW revised the manuscript. All authors read and approved the final manuscript.

## Funding

This work was supported by the National Natural ScienceFoundation of China (grant number 32070701 to QW and grant number 32200976 to XS), Startup Funds from Tianjin Medical University (grant number 115004/000012 and 11601502/DW0114 to QW), the Science & Technology Development Fund of Tianjin Education Commission for Higher Education (grant number 2018KJ085 to XS), and the National Laboratory of Biomacromolecules (grant number 2020kf11 to QW).

## Conflict of interest

The authors declare that the research was conducted in the absence of any commercial or financial relationships that could be construed as a potential conflict of interest.

## Publisher’s note

All claims expressed in this article are solely those of the authors and do not necessarily represent those of their affiliated organizations, or those of the publisher, the editors and the reviewers. Any product that may be evaluated in this article, or claim that may be made by its manufacturer, is not guaranteed or endorsed by the publisher.

## Supplementary material

Supplementary Figure 1 Mannose inhibits blood-stage rodent *Plasmodium* growth in mice and drug-resistant *Plasmodium falciparum* growth *in vitro*. **(A, B)** Mannose (20%) inhibited *P. berghei* ANKA growth in BALB/c mice (n = 5/group). Drinking water or mannose were supplemented by oral gavage every other day from 1, 2, or 5 days post-infection (p.i.) or 10 days prior to parasite infection. CTRL vs. Mannose (+) day 2 p.i.: *P* < 0.0001; CTRL vs. Mannose (+) day 5 p.i.: *P* < 0.05; Mannose (+) day 2 p.i. vs. Mannose (+) day 5 p.i.: *P* < 0.001; CTRL vs. Mannose (+) day 1 p.i.: *P* < 0.0001; CTRL vs. Mannose (+) 10 days prior to infection: *P* < 0.0001; Mannose (+) 10 days prior to infection vs. Mannose (+) day 1 p.i.: n.s. **(C)** D-mannose inhibited blood-stage *P. falciparum* Dd2 growth *in vitro*. CTRL vs. Mannose (20/30/40/50 mM): *P* < 0.0001. **(D)** D-mannose inhibited blood-stage *P. falciparum* 803 growth *in vitro*. CTRL vs. Mannose (20/30/40/50 mM): *P* < 0.0001.* P. falciparum* Dd2 and 803 were cultured *in vitro* with a starting parasitemia of 1% on day 0 (n = 3/group). The indicated dose of D-mannose was added to the media for 1 hour from day 0 and parasitemia measured daily before mannose treatment. Data are presented as mean ± SD and analyzed by two-way ANOVA with Tukey’s multiple comparisons test.

Supplementary Figure 2 Mannose cannot induce oxidative stress and eryptosis in *Plasmodium*-infected erythrocytes (iRBCs). **(A)** Mannose did not cause superoxide production in *P. berghei*-iRBCs and uninfected rat RBCs. Representative FACS plots and quantitative superoxide production was determined by DHE in iRBCs and RBCs (n = 3/group). Menadione was used as a positive control. **(B)** Mannose did not induce mitochondrial superoxide production in *P. berghei*-iRBCs. Representative FACS plots and quantitative superoxide production was determined by MitoSOX Red in iRBCs (n = 3/group). H_2_O_2_ was used as a positive control. **(C)** Mannose did not induce oxidative stress in cultured *P. falciparum* 3D7-iRBCs. Quantitative superoxide production was measured by DHE and MitoSOX Red in iRBCs (n = 3/group). **(D)** Mannose did not induce eryptosis in normal rat RBCs and *P. berghei*-iRBCs. Representative FACS plots and quantitative analysis of apoptosis in RBCs and iRBCs labeled with Annexin V-FITC are shown (n = 3/group). Vitamin C-treated cells were used as positive controls. **(E)** Mannose did not induce eryptosis in *P. falciparum* 3D7-iRBCs as determined by Annexin V-FITC (n = 3/group). (F) Mannose did not induce apoptosis in *P. berghei* parasites detected by TUNEL assay (n = 3/group). DNase I-treated cells were used as positive controls. All data are presented as mean ± SD, and differences between drug-treated RBCs/iRBCs and non-treated RBCs/iRBCs (CTRL) were analyzed by the Kruskal-Wallis ANOVA or one-way ANOVA.

Supplementary Figure 3 Mannose treatment is well tolerated in naive uninfected mice. **(A, B)** D-mannose treatment influenced body weight and food intake in uninfected C57BL/6 mice compared to drinking water-treated mice (CTRL) or non-treated mice (Blank) (n = 5/group). Two-way ANOVA with Tukey’s multiple comparisons test was performed in (A) (Mannose vs. CTRL: *P* < 0.0001, Mannose vs. Blank: *P* < 0.001). **(C, D)** Daily treatment with 20% D-mannose for 20 days did not impair liver or kidney function in uninfected BALB/c mice (n = 3-5/group). Kruskal-Wallis ANOVA was performed. All data are presented as mean ± SD.

Supplementary Figure 4 Mannose treatment plays different roles in splenic macrophage levels in *P. berghei-*infected and uninfected C57BL/6 mice. **(A, B)** Representative flow cytometry dot plots of F4/80^+^ CD11b^+^ macrophages in CD45^+^ splenocytes from C57BL/6 mice in . **(C, D)** Representative flow cytometry dot plots of F4/80^+^ CD11b^+^ macrophages in CD45^+^ splenocytes from the uninfected C57BL/6 mice on day 4 **(C)** and day 7 **(D)**. **(E)** Spleen weights for the uninfected C57BL/6 mice on day 4 (n = 5/group). (F) The frequency and number of splenic macrophages (CD45^+^ F4/80^+^ CD11b^+^) in the uninfected C57BL/6 mice on day 4 (n = 5/group). **(G)** Quantification of the ratio of M1 macrophages (MHC II^+^) to total splenic macrophages in the uninfected mice on day 4. **(H)** Phagocytic capacity of splenic macrophages from the uninfected mice on day 4. The percentage of phagocytosing FITC^+^ macrophages and MFI values are shown. Data are presented as mean ± SD. Analyses were carried out by one-way ANOVA followed by Tukey’s multiple comparison test.

Supplementary Figure 5 Mannose treatment does not influence the splenic macrophage population in *P. berghei-*infected BALB/c mice. **(A)** Parasitemia of *P. berghei-*infected BALB/c mice (n = 5/group). Two-way ANOVA with Tukey’s multiple comparisons test was used to analyze differences in parasitemia between the control and mannose-treated groups. **(B)** Spleen weight in BALB/c mice (n = 5/group) 4 and 7 days post-infection (p.i.). The weights of spleens harvested from uninfected mice (n = 5/group) are shown as blank controls. **(C)** Representative flow cytometry dot plots of F4/80^+^ CD11b^+^ macrophages in CD45^+^ splenocytes from BALB/c mice 4 days p.i. (n = 5/group) and the uninfected mice (n = 5/group). **(D)** The frequency and number of splenic macrophages in **(C)**. **(E)** Representative flow cytometry dot plots of F4/80^+^ CD11b^+^ macrophages in CD45^+^ splenocytes from BALB/c mice 7 days p.i. (n = 5/group) and the uninfected mice (n = 5/group). **(F)** The frequency and number of splenic macrophages in **(E)**. Data are presented as mean ± SD. Analyses were carried out by one-way ANOVA followed by Tukey’s multiple comparison test.

Supplementary Figure 6 Mannose treatment suppresses T-cell migration into the brain. **(A, B)** Representative flow cytometry dot plots for showing the CD4^+^ T cells **(A)** and CD8^+^ T cells **(B)** in peripheral blood mononuclear cells from uninfected and infected C57BL/6 mice 7 days p.i. **(C, D)** Representative flow cytometry dot plots for showing the CD4^+^ T cells **(C)** and CD8^+^ T cells **(D)** in brain mononuclear cells from uninfected and infected C57BL/6 mice 7 days p.i. **(E, F)** Treatment with mannose significantly decreased IFN-γ and TNF-α mRNA expression in the brains of infected C57BL/6 mice 7 days p.i. (n = 6-8/group).
