# Supplementary figures and images for: Mannose inhibits Plasmodium parasite growth and cerebral malaria development via regulation of host immune responses
Source: Front Immunol. 2022 Sep 23;13:859228. doi: 10.3389/fimmu.2022.859228 (PMC9546034; doi:10.3389/fimmu.2022.859228)

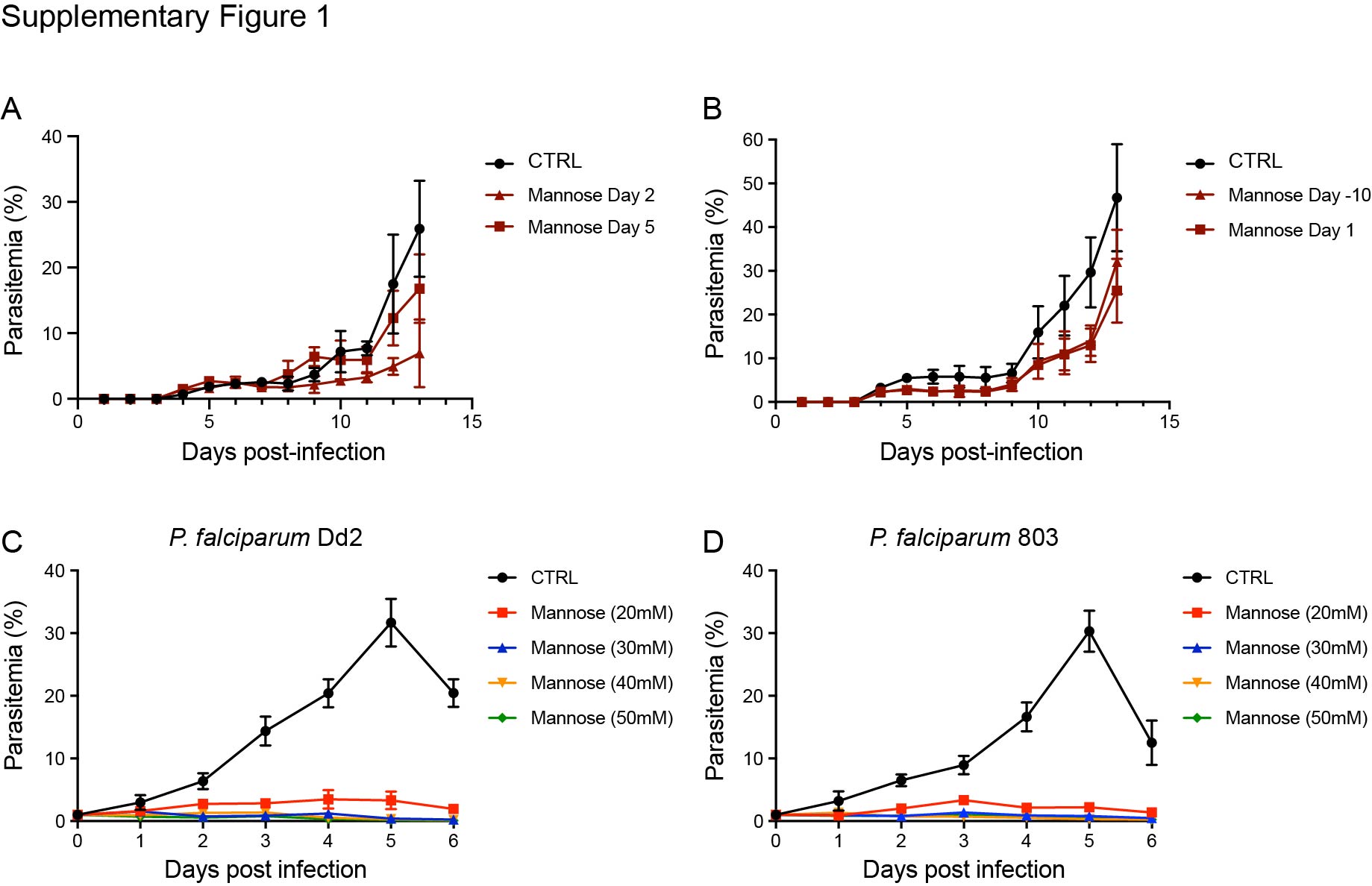

Supplement: Supplementary Figure 1 — Mannose inhibits blood-stage rodent Plasmodium growth in mice and drug-resistant Plasmodium falciparum growth in vitro. (A, B) Mannose (20%) inhibited P. berghei ANKA growth in BALB/c mice (n = 5/group). Drinking water or mannose were supplemented by oral gavage every other day from 1, 2, or 5 days post-infection (p.i.) or 10 days prior to parasite infection. CTRL vs. Mannose (+) day 2 p.i.: P < 0.0001; CTRL vs. Mannose (+) day 5 p.i.: P < 0.05; Mannose (+) day 2 p.i. vs. Mannose (+) day 5 p.i.: P < 0.001; CTRL vs. Mannose (+) day 1 p.i.: P < 0.0001; CTRL vs. Mannose (+) 10 days prior to infection: P < 0.0001; Mannose (+) 10 days prior to infection vs. Mannose (+) day 1 p.i.: n.s. (C) D-mannose inhibited blood-stage P. falciparum Dd2 growth in vitro. CTRL vs. Mannose (20/30/40/50 mM): P < 0.0001. (D) D-mannose inhibited blood-stage P. falciparum 803 growth in vitro. CTRL vs. Mannose (20/30/40/50 mM): P < 0.0001. P. falciparum Dd2 and 803 were cultured in vitro with a starting parasitemia of 1% on day 0 (n = 3/group). The indicated dose of D-mannose was added to the media for 1 hour from day 0 and parasitemia measured daily before mannose treatment. Data are presented as mean ± SD and analyzed by two-way ANOVA with Tukey’s multiple comparisons test. [file Image_1.jpeg]

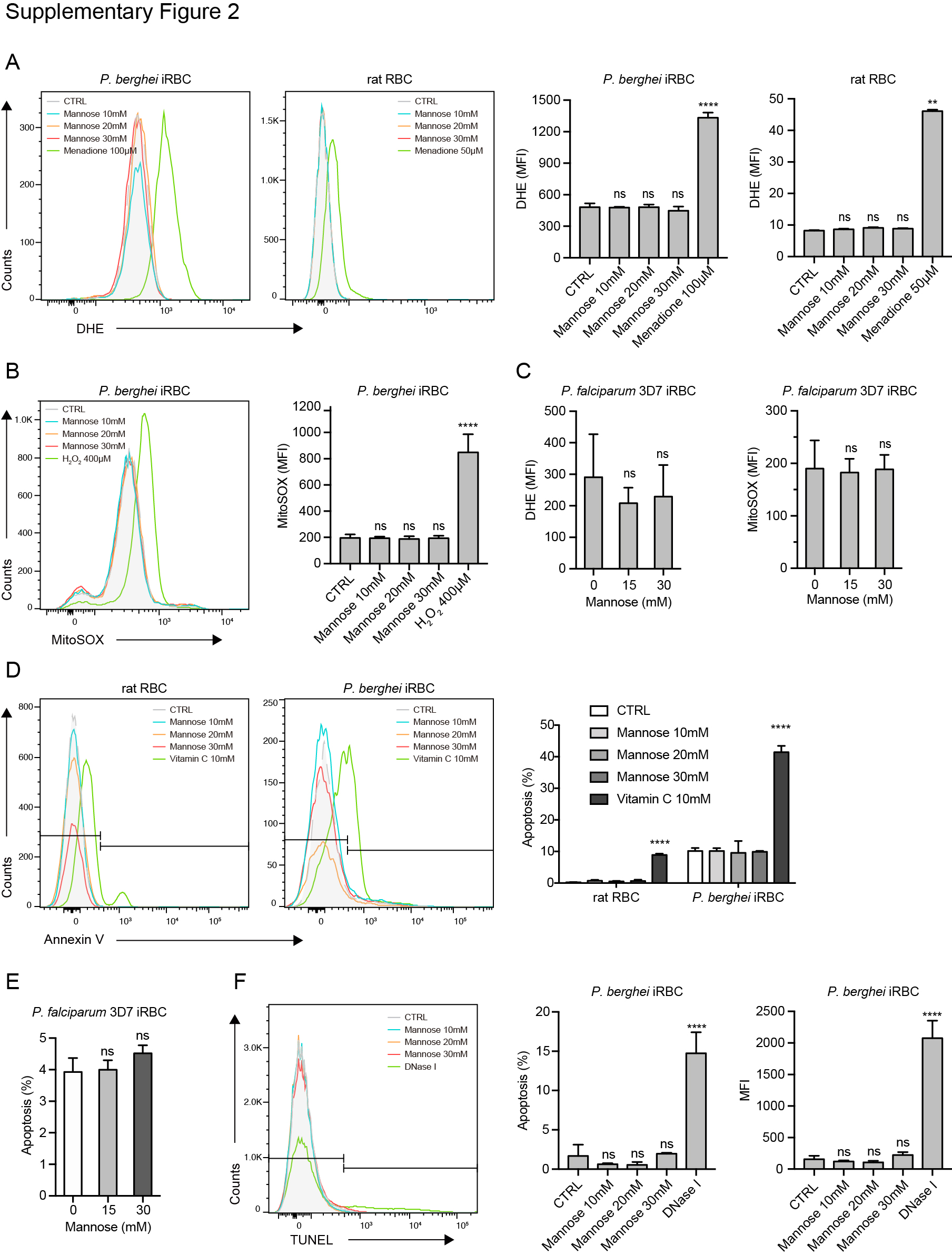

Supplement: Supplementary Figure 2 — Mannose cannot induce oxidative stress and eryptosis in Plasmodium-infected erythrocytes (iRBCs). (A) Mannose did not cause superoxide production in P. berghei-iRBCs and uninfected rat RBCs. Representative FACS plots and quantitative superoxide production was determined by DHE in iRBCs and RBCs (n = 3/group). Menadione was used as a positive control. (B) Mannose did not induce mitochondrial superoxide production in P. berghei-iRBCs. Representative FACS plots and quantitative superoxide production was determined by MitoSOX Red in iRBCs (n = 3/group). H2O2 was used as a positive control. (C) Mannose did not induce oxidative stress in cultured P. falciparum 3D7-iRBCs. Quantitative superoxide production was measured by DHE and MitoSOX Red in iRBCs (n = 3/group). (D) Mannose did not induce eryptosis in normal rat RBCs and P. berghei-iRBCs. Representative FACS plots and quantitative analysis of apoptosis in RBCs and iRBCs labeled with Annexin V-FITC are shown (n = 3/group). Vitamin C-treated cells were used as positive controls. (E) Mannose did not induce eryptosis in P. falciparum 3D7-iRBCs as determined by Annexin V-FITC (n = 3/group). (F) Mannose did not induce apoptosis in P. berghei parasites detected by TUNEL assay (n = 3/group). DNase I-treated cells were used as positive controls. All data are presented as mean ± SD, and differences between drug-treated RBCs/iRBCs and non-treated RBCs/iRBCs (CTRL) were analyzed by the Kruskal-Wallis ANOVA or one-way ANOVA. [file Image_2.jpeg]

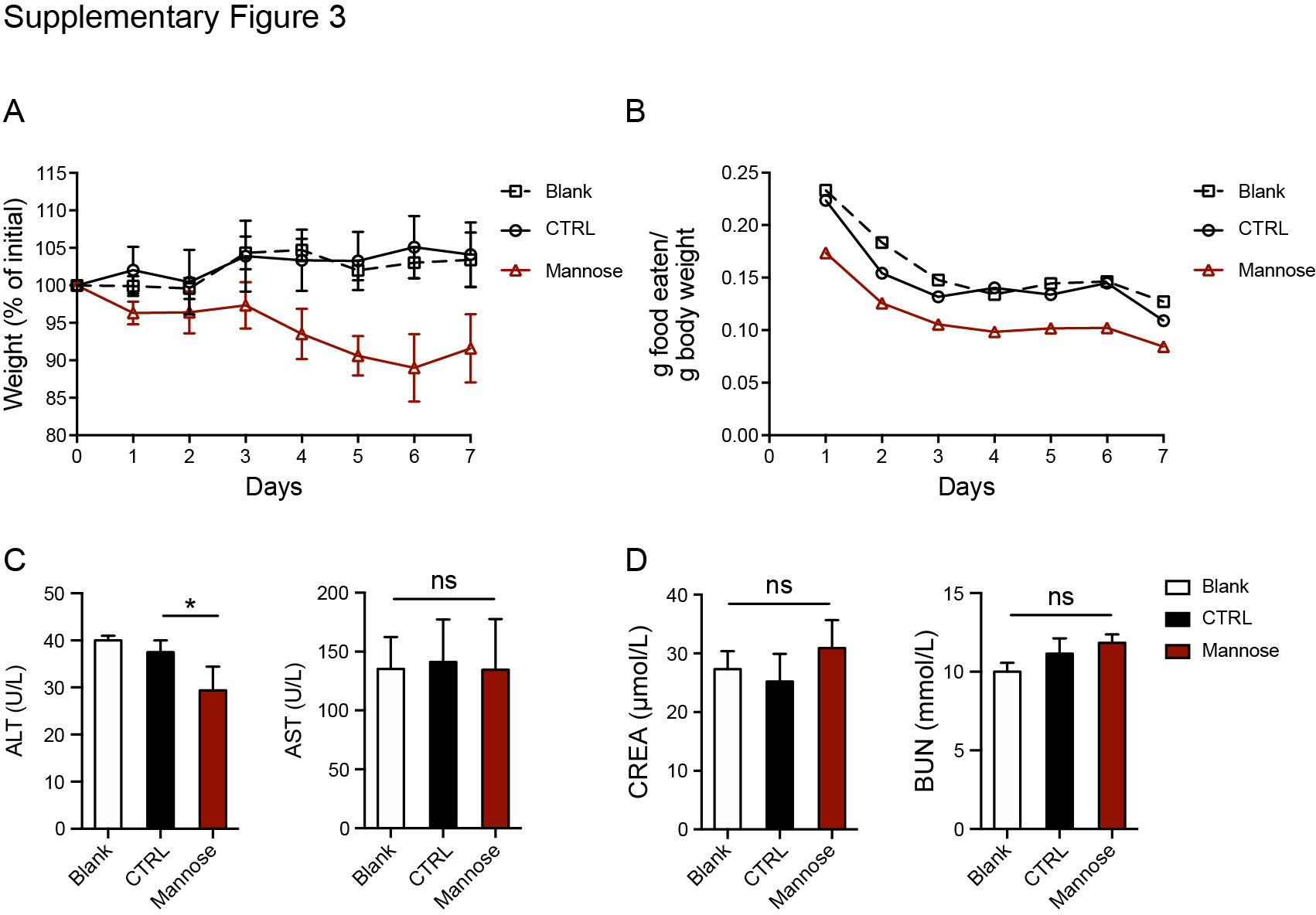

Supplement: Supplementary Figure 3 — Mannose treatment is well tolerated in naive uninfected mice. (A, B) D-mannose treatment influenced body weight and food intake in uninfected C57BL/6 mice compared to drinking water-treated mice (CTRL) or non-treated mice (Blank) (n = 5/group). Two-way ANOVA with Tukey’s multiple comparisons test was performed in (A) (Mannose vs. CTRL: P < 0.0001, Mannose vs. Blank: P < 0.001). (C, D) Daily treatment with 20% D-mannose for 20 days did not impair liver or kidney function in uninfected BALB/c mice (n = 3-5/group). Kruskal-Wallis ANOVA was performed. All data are presented as mean ± SD. [file Image_3.jpeg]

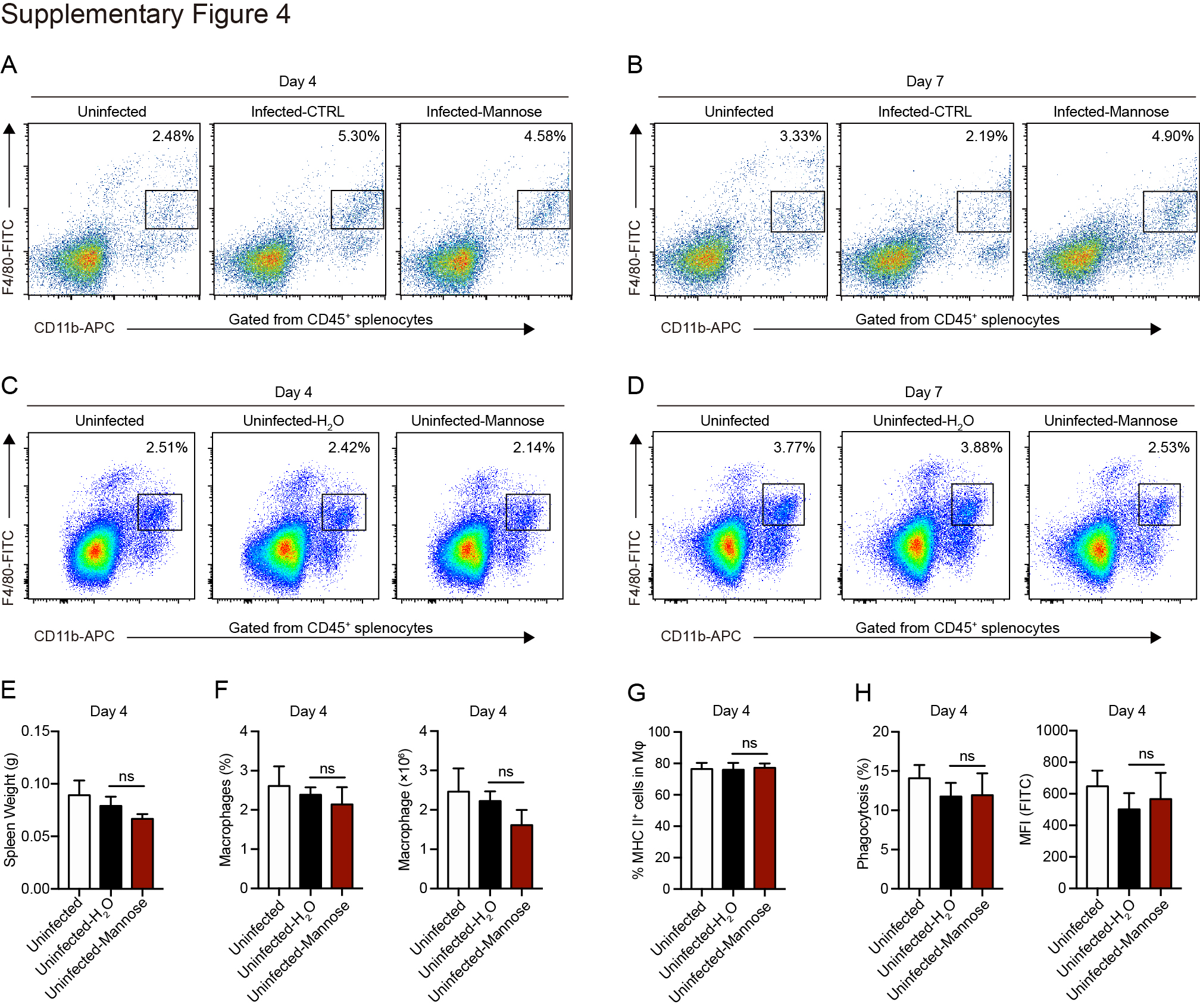

Supplement: Supplementary Figure 4 — Mannose treatment plays different roles in splenic macrophage levels in P. berghei-infected and uninfected C57BL/6 mice. (A, B) Representative flow cytometry dot plots of F4/80+ CD11b+ macrophages in CD45+ splenocytes from C57BL/6 mice in . (C, D) Representative flow cytometry dot plots of F4/80+ CD11b+ macrophages in CD45+ splenocytes from the uninfected C57BL/6 mice on day 4 (C) and day 7 (D). (E) Spleen weights for the uninfected C57BL/6 mice on day 4 (n = 5/group). (F) The frequency and number of splenic macrophages (CD45+ F4/80+ CD11b+) in the uninfected C57BL/6 mice on day 4 (n = 5/group). (G) Quantification of the ratio of M1 macrophages (MHC II+) to total splenic macrophages in the uninfected mice on day 4. (H) Phagocytic capacity of splenic macrophages from the uninfected mice on day 4. The percentage of phagocytosing FITC+ macrophages and MFI values are shown. Data are presented as mean ± SD. Analyses were carried out by one-way ANOVA followed by Tukey’s multiple comparison test. [file Image_4.jpeg]

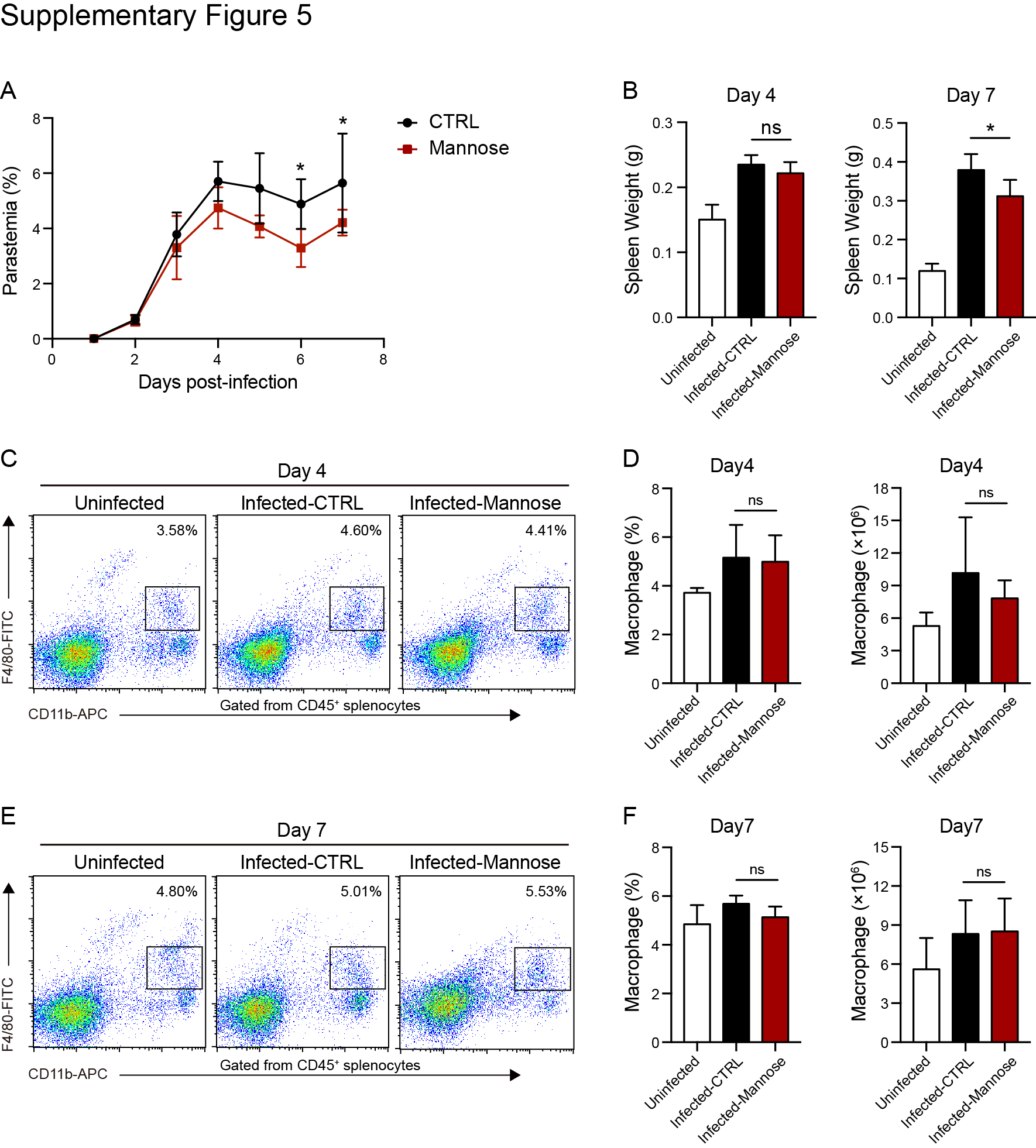

Supplement: Supplementary Figure 5 — Mannose treatment does not influence the splenic macrophage population in P. berghei-infected BALB/c mice. (A) Parasitemia of P. berghei-infected BALB/c mice (n = 5/group). Two-way ANOVA with Tukey’s multiple comparisons test was used to analyze differences in parasitemia between the control and mannose-treated groups. (B) Spleen weight in BALB/c mice (n = 5/group) 4 and 7 days post-infection (p.i.). The weights of spleens harvested from uninfected mice (n = 5/group) are shown as blank controls. (C) Representative flow cytometry dot plots of F4/80+ CD11b+ macrophages in CD45+ splenocytes from BALB/c mice 4 days p.i. (n = 5/group) and the uninfected mice (n = 5/group). (D) The frequency and number of splenic macrophages in (C). (E) Representative flow cytometry dot plots of F4/80+ CD11b+ macrophages in CD45+ splenocytes from BALB/c mice 7 days p.i. (n = 5/group) and the uninfected mice (n = 5/group). (F) The frequency and number of splenic macrophages in (E). Data are presented as mean ± SD. Analyses were carried out by one-way ANOVA followed by Tukey’s multiple comparison test. [file Image_5.jpeg]

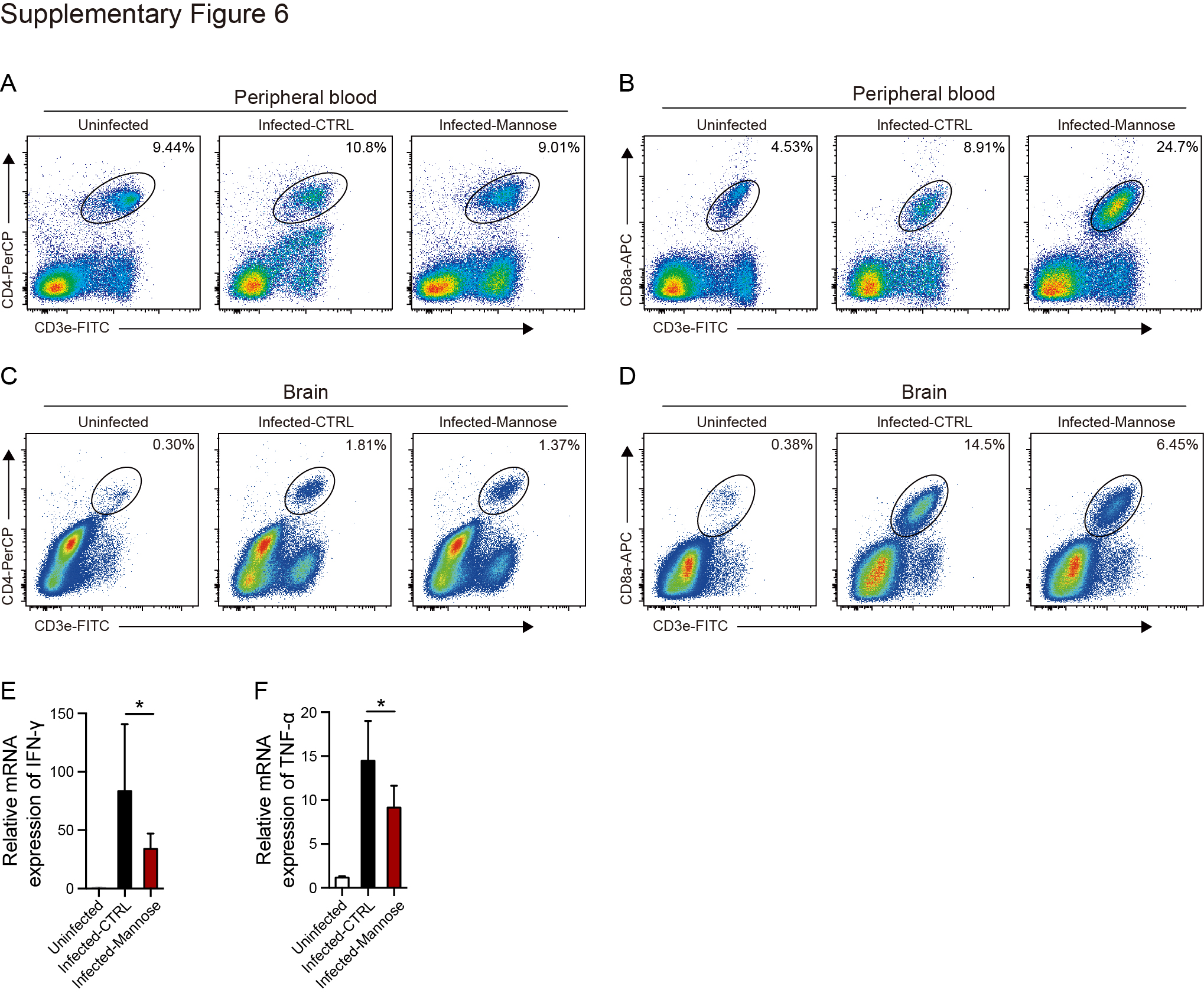

Supplement: Supplementary Figure 6 — Mannose treatment suppresses T-cell migration into the brain. (A, B) Representative flow cytometry dot plots for showing the CD4+ T cells (A) and CD8+ T cells (B) in peripheral blood mononuclear cells from uninfected and infected C57BL/6 mice 7 days p.i. (C, D) Representative flow cytometry dot plots for showing the CD4+ T cells (C) and CD8+ T cells (D) in brain mononuclear cells from uninfected and infected C57BL/6 mice 7 days p.i. (E, F) Treatment with mannose significantly decreased IFN-γ and TNF-α mRNA expression in the brains of infected C57BL/6 mice 7 days p.i. (n = 6-8/group). [file Image_6.jpeg]
